# Multi-wavelength emission through self-induced second-order wave-mixing processes from a Nd^3+^ doped crystalline powder random laser

**DOI:** 10.1038/srep13816

**Published:** 2015-09-03

**Authors:** André L. Moura, Vladimir Jerez, Lauro J. Q. Maia, Anderson S. L. Gomes, Cid B. de Araújo

**Affiliations:** 1 Grupo de Física da Matéria Condensada, Núcleo de Ciências Exatas – NCEx, Campus Arapiraca, Universidade Federal de Alagoas, 57309-005, Arapiraca-AL, Brazil; 2 Departamento de Física, Universidade Federal de Pernambuco, 50670-901, Recife-PE, Brazil; 3 CIBAS, Universidad de Santander, Bucaramanga, Colombia; 4 Grupo Física de Materiais, Instituto de Física, Universidade Federal de Goiás, 74001-970, Goiânia-GO, Brazil

## Abstract

Random lasers (RLs) based on neodymium ions (Nd^3+^) doped crystalline powders rely on multiple light scattering to sustain laser oscillation. Although Stokes and anti-Stokes Nd^3+^ RLs have been demonstrated, the optical gain obtained up to now was possibly not large enough to produce self-frequency conversion. Here we demonstrate self-frequency upconversion from Nd^3+^ doped YAl_3_(BO_3_)_4_ monocrystals excited at 806 nm, in resonance with the Nd^3+^ transition ^4^I_9/2_ → ^4^F_5/2_. Besides the observation of the RL emission at 1062 nm, self-converted second-harmonic at 531 nm, and self-sum-frequency generated emission at 459 nm due to the RL and the excitation laser at 806 nm, are reported. Additionally, second-harmonic of the excitation laser at 403 nm was generated. These results exemplify the first multi-wavelength source of radiation owing to nonlinear optical effect in a Nd^3+^ doped crystalline powder RL. Contrary to the RLs based on dyes, this multi-wavelength light source can be used in photonic devices due to the large durability of the gain medium.

The optical properties of a medium can be described by the polarization vector that is written in the component form as 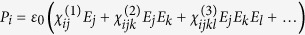 (ref. 1), where the first, second and third terms on the right describe the linear, second and third-order nonlinear responses of the material, respectively. The second-order susceptibility, 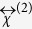, present in noncentrosymmetric materials, is responsible for optical rectification (OR), linear electrooptic effect (LEE), second-harmonic generation (SHG), sum- and difference-frequency generation (SFG and DFG, respectively). SHG, SFG and DFG are used for frequency conversion, OR is an optically induced DC electric field, and the LEE is used to modulate the linear refractive index of a medium by an applied electric field.

The observation of the above-mentioned effects does not require resonance excitation of particular transitions in the nonlinear medium with redistribution of population between excited states. On the other hand, the operation of lasers requires population inversion among energy levels of the active medium in order to obtain an optical amplification that compensates the losses in the optical cavity. Indeed, due to the large intensity generated in the lasing process, internal nonlinear frequency conversion can be observed in some laser systems that present large optical gain. The laser generation and its simultaneous internal nonlinear conversion, known as self-frequency conversion (SFC), are well-understood in conventional lasers, i.e., lasers with mirrors cavity. Examples are the generation of neodymium ion (Nd^3+^) laser emission at 1.06 μm and its conversion to 0.53 μm (ref. 2 and 3) and 1.18 μm (ref. 4), which correspond to the second-harmonic and the Stokes stimulated Raman scattering, respectively. More recently self-frequency doubling was exploited in waveguide lasers^5^ and high efficient operation of CW upconversion laser^6^. Besides this fascinating physical phenomenon, it is interesting from the applied point of view to exploit processes of frequency conversion inside a unique medium.

Observation of SFC has not been reported for random lasers (RLs), which constitute a very interesting class of laser systems based on the multiple scattering of light inside or outside an amplifying medium. RLs were theoretically predicted in 1968 (ref. 7), and have attracted much attention in the last two decades since its first unambiguous demonstration^8^, which used a colloidal solution of dye and TiO_2_ scatterers. RLs have been shown to operate from a large variety of systems as distinct as ZnO nanostructures^9^, powders of micron-sized particles doped with rare earth ions^10^, human tissues^11^, cold atoms^12^, colloidal doped photonics crystal fibers^13^, combined Rayleigh scattering and Raman amplification in conventional optical fibers^14^, and Raman gain in barium sulphate powder^15^. Earlier works, including RLs fundamentals and applications, have been recently reviewed^10^,^16^,^17^,^18^,^19^. Nowadays, one of the research trends is the study of new physical phenomena in RLs, such as its spin glass behavior^20^ or Levy-like statistics in the intensity fluctuations^21^. Recent applications include the use of RLs as speckle-free imaging sources^22^ or for chemical identification of powders^23^.

RLs based on Nd^3+^ are well-known and characterized^10^. Generally, powders of micro-sized crystalline or glassy particles doped with Nd^3+^ are excited in resonance with the transition ^4^I_9/2_ → ^4^F_5/2_ (around 800 nm) and RL operation due to the transition ^4^F_3/2_ → ^4^I_11/2_ is observed at 1.06 μm. In the present communication, anti-Stokes SFC in a Nd^3+^ RL is reported and novel features are revealed. Powders constituted of grains of Nd^3+^:YAl_3_(BO_3_)_4_ monocrystals were excited at 806 nm readily producing the RL emission at 1062 nm. Thereupon, self-frequency upconversion processes were observed arising from SHG of the 1062 nm RL emission at 531 nm, and SFG due to wave-mixing between the pump and the RL emission generating light at 459 nm. Furthermore, SHG of the excitation light beam was observed at 403 nm. To the best of our knowledge, this is the first report of SFC in RLs which illustrates the possibility of obtaining novel multi-wavelength sources based on parametric generation in RL, leading to photon sources from the UV to the infrared.

## Results

### Experimental details

**Figure 1:**
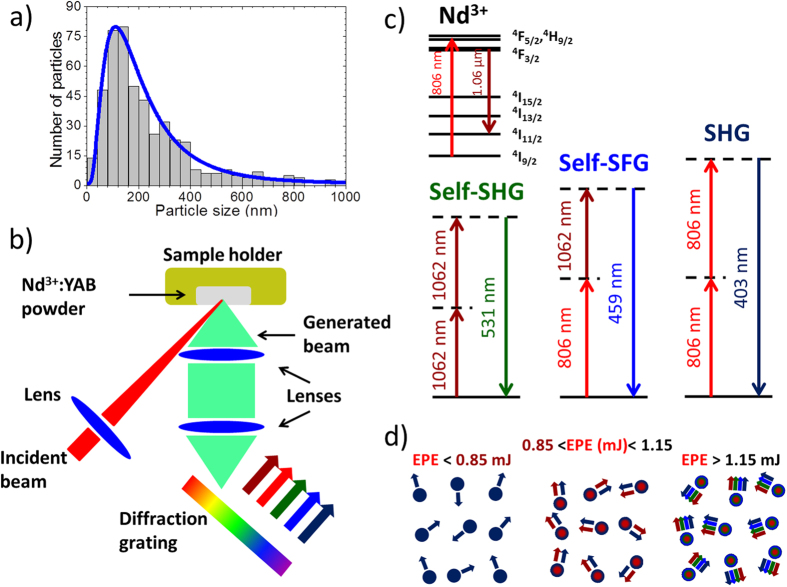
Size distribution of the powder grains, optical experimental representation, pictorial description of the internal processes in the Nd:YAB powder, and energy level diagrams of the internal processes. (**a**) Size distribution of the Nd:YAB powder grains. (**b**) Schematic of the experimental setup shown the disordered powder excited at 806 nm emitting at 1062, 531, 459, and 403 nm. (**c**) Energy level diagrams associated to the frequency conversion processes. (**d**) Processes inside the Nd:YAB grains for different ranges of excitation pulse energy: excitation second-harmonic generation (SHG) (dark blue), random laser (RL) emission (dark red), self-SHG (green), and self-sum-frequency generation (Self-SFG) (blue).

Crystalline powders of Nd_0.04_Y_0.96_Al_3_(BO_3_)_4_ (labeled here as Nd:YAB) with particle diameters size ranging from 20 to 600 nm in which the size distribution, shown in Fig. 1a, is centered at ~173 nm were used. The Nd^3+^ concentration was ~2.2 × 10^20^ ions/cm^3^ and they were prepared by the polymeric precursor method.

A simplified representation of the experiment is given in Fig. 1b. The optical experiments were conducted with the powder excited by an Optical Parametric Oscillator (OPO) pumped by a Q-switched Nd:YAG laser (7 ns, 10 Hz). The powder was placed in a sample holder and gently pressed into a uniform disc region. The light beam from the OPO was focused on the sample by a 10 cm focal length lens. Unless specified, the illuminated area was 2.1 mm^2^. The excitation wavelength was chosen to 806 nm to optimize the fluorescence signal at 1.06 μm. The energy level diagrams in Fig. 1c illustrate the nonlinear processes corresponding to the multicolor emission. A draw representing the multi-wavelength generation is presented in Fig. 1d for different values of the excitation pulse energy (EPE).

### Nd^3+^ random laser

**Figure 2:**
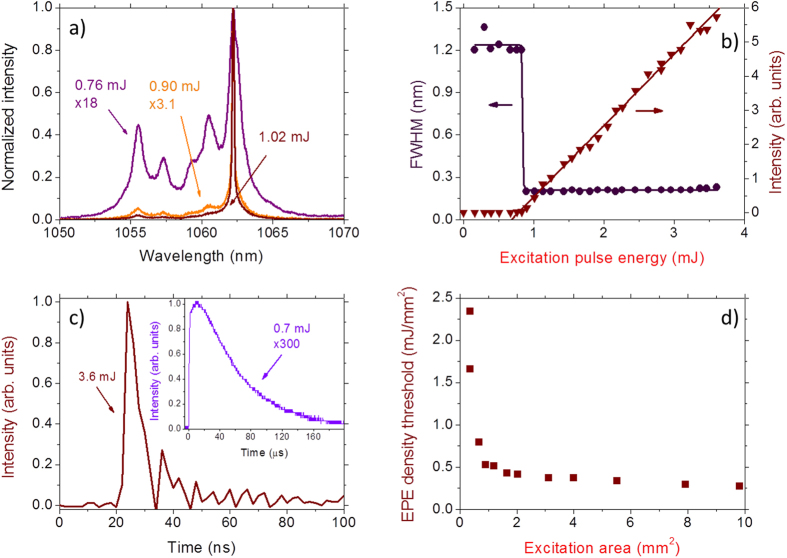
Threshold behavior of the 1062 nm random laser. (**a**) Normalized spectra of the Nd^3+^ transition ^4^F_3/2_ → ^4^I_11/2_ for different excitation pulse energies. (**b**) Full width at half maximum and normalized intensity dependence with the excitation pulse energy (EPE) of the band centered at 1062 nm. The curves shown are only to guide the eyes. (**c**) Temporal response of the system for EPE smaller and larger than the RL threshold (0.85 mJ). (**d**) EPE density threshold dependence with the excitation area.

Exciting the Nd:YAB sample at 806 nm (Nd^3+^ transition: ^4^I_9/2_ → ^4^F_5/2_) gave rise to the RL emission at 1062 nm (Nd^3+^ transition: ^4^F_3/2_ → ^4^I_11/2_) and the results are summarized in Fig. 2. Figure 2a shows the collected spectrum for three different EPE: below, close and above the threshold. The abrupt change in the RL intensity versus the EPE slope and the spectral narrowing of the RL emission at 1062 nm as the EPE was increased are unveiled in Fig. 2b, from where a RL threshold of 0.85 mJ was determined. Similar to references 24 and 25, the RL spectrum did not show the spikes that are characteristic of resonant feedback, and therefore the RL is operating in the nonresonant feedback regime. The RL threshold was also verified by measuring the temporal behavior of the emitted light shown in Fig. 2c where, for EPE of 0.70 mJ, therefore below the threshold, an exponential decay with lifetime of the order of 80 μs is observed for the signal centered at 1.06 μm. Increasing the EPE above the threshold, also shown in Fig. 2c for an EPE of 3.6 mJ, a pulse with about 10 nanoseconds duration is observed, limited by the detector resolution, clearly corroborating the RL action. The temporal behavior of Nd^3+^ RL has been already investigated in detail in ref. 26.

The EPE density threshold, presented in Fig. 2d, is dependent on the excitation area as reported for other RLs^10^,^25^. This behavior is due to a balance between the volume effectively excited and the pathways of the incident and the emitted photons inside the gain region^25^. Small excited volumes require high EPE density to obtain RL emission, because the time interval elapsed by photons inside the gain region and the pathway are small. The present results on the 1062 nm RL properties are comparable to the RL behavior observed using micrometric (~4 μm) grains with the same chemical composition^24^. However, further multi-wavelength emission not reported before, was clearly observed and characterized in the present work.

### Frequency upconversions

**Figure 3:**
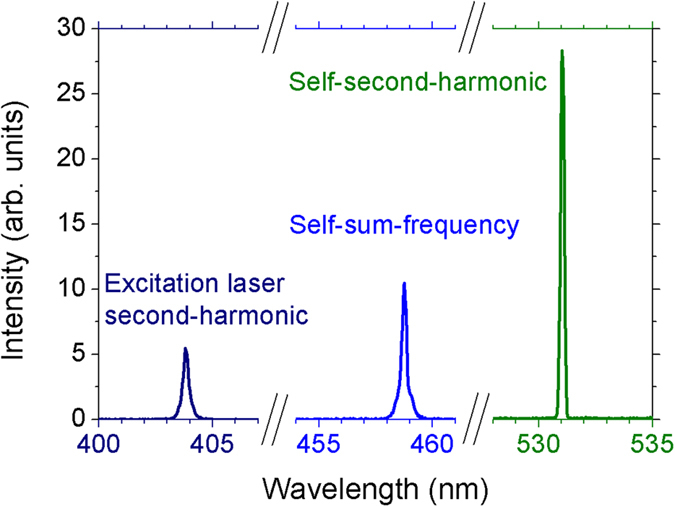
Upconversion spectrum. From left to right, the signals are the second-harmonic of the excitation OPO wavelength at 806 nm, the self-sum-frequency due to the OPO beam and the 1062 nm RL, and the self-second-harmonic of the 1062 nm RL, respectively.

Figure 3 shows the observed frequency upconversion signals at 403, 459 and 531 nm for an EPE of 3.6 mJ. The relative amplitude of the signals was corrected by the CCD spectral response. The emissions in the blue/green region could be seen by naked eye and were not due to any Nd^3+^ transition as in ref. 27. In fact, they are associated to the second-order nonlinearity of the noncentrosymmetric Nd:YAB crystal grains. Just as indicated in Fig. 1c, the deep blue (403 nm) is the second-harmonic of the excitation OPO wavelength (806 nm), and therefore it corresponds to SHG inside the crystalline grains. The blue emission at 459 nm is due to the SFG between the excitation beam at 806 nm and the RL at 1062 nm, self-induced in this open cavity system; finally, the green emission at 531 nm is the SHG of the 1062 nm RL, also self-induced.

## Discussion

**Figure 4:**
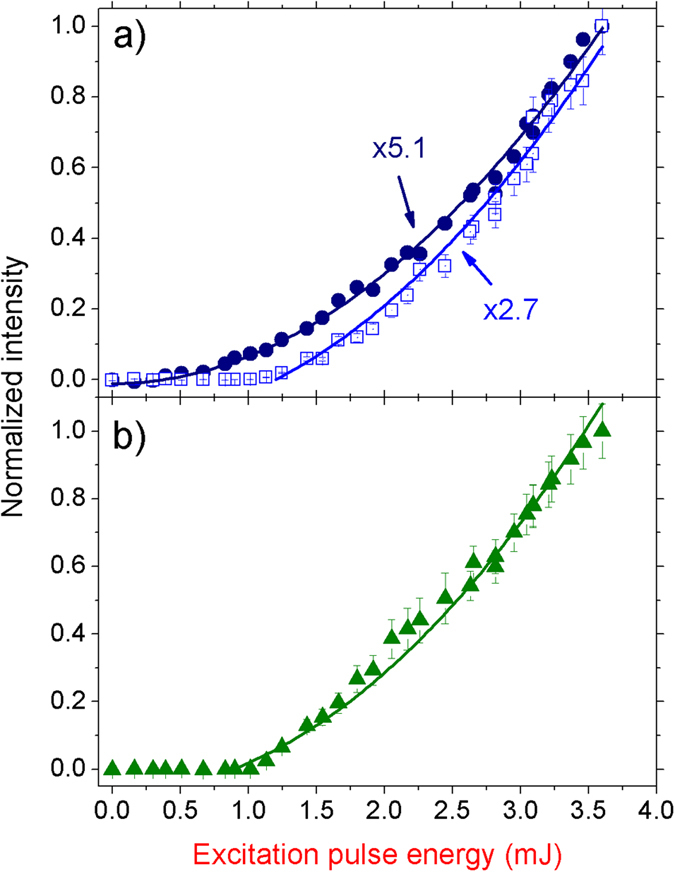
Intensities of the frequency upconversion signals versus the excitation pulse energy. (**a**) Second-harmonic emission at 403 nm (closed circles) and self-sum-frequency emission at 459 nm (open squares). (**b**) Self-second-harmonic emission at 531 nm. The curves shown in (a) and (**b**) are quadratic fittings of the data.

All of the observed upconverted signals were originated from parametric processes and, consequently, their bandwidths are narrow, and measured to be ~0.2 nm, close to the instrumental resolution. The nonlinear nature of these transitions is evidenced in Figure 4 where their relative intensity dependences with the EPE together with the corresponding quadratic fittings are plotted. Notice that the emission at 403 nm starts to grow in intensity as soon as there is enough power to be detected, and therefore does not present any threshold behavior. On the other hand, the emissions at 459 and 531 nm clearly present an EPE critical value of 1.15 mJ, which is larger than the 1062 nm RL threshold (0.85 mJ). This is an expected behavior, since of the second-harmonic signal at 403 nm is an externally pumped process, whereas the emissions at 459 and 531 nm require the existence of the RL emission with a minimum power level beyond its threshold value for the self-upconverted emissions be detected. Figure 1d summarizes the EPE threshold values of the generated emissions, where for EPE <0.85 mJ, only the second-harmonic of the excitation OPO at 403 nm was observed. Increasing the EPE in the range from 0.85 mJ up to 1.15 mJ, the emission at 403 nm and the RL at 1062 nm were detected. Finally, in the situations of EPE >1.15 mJ, besides the 1062 nm emission, all upconverted signals were observed.

In summary, RL emission at 1062 nm from crystalline grains of Nd^3+^:YAl_3_(BO_3_)_4_ was demonstrated together with parametric processes as self-frequency doubling at 531 nm, and self-sum-frequency generation at 459 nm due to wave-mixing of the excitation laser (806 nm) and the RL emission (1062 nm). This first time demonstration of multi-wavelength parametric generation triggered by the RL generation opens up yet a new avenue for investigation of RL based photon sources. Even though the visible parametric emissions were strong enough to be seen by naked eyes, the overall efficiency can be improved using particles with optimized size and Nd^3+^ concentration, inasmuch as second-order nonlinear processes in powders are dependent on the relation between the particles’ size and the coherence length^28^, and the RL performance depends on the Nd^3+^ concentration^10^,^24^, respectively. In principle, tunable parametric generation leading to ultraviolet, visible, infrared, and mid-infrared sources in noncentrosymmetric monocrystals with appropriate energy bandgap can be observed. Procedures such as shaping the spatial intensity distribution of the excitation source to obtain directional RL emission^29^ and single-mode operation^30^, already applied to single wavelength RLs, can be extended to these proposed parametric generators.

## Methods

### Powder preparation

The Nd:YAB powder was obtained with citric acid (C_5_O_7_H_8_, Sigma-Aldrich) as a complexing agent, d-sorbitol (C_6_O_6_H_14_, Sigma-Aldrich 98%) as polymerizing agent, boric acid (H_3_BO_3_, Ecibra 99.5%), aluminum nitrate nonahydrate (Al(NO_3_)_3_.9H_2_O, Vetec 98%), yttrium nitrate hexahydrate (Y(NO_3_)_3_.6H_2_O, Sigma-Aldrich 99.8%), and neodymium hexahydrate (Nd(NO_3_)_3_.6H_2_O, Sigma-Aldrich 99.8%) as precursors for B, Al, Y, and Nd, respectively. The synthesis of the material was achieved by dissolving aluminum, yttrium, and neodymium nitrates in an aqueous solution of citric acid at room temperature. This solution was added to another solution of d-sorbitol and boric acid dissolved previously in water. The obtained solution was annealed at 150 ^o^C in an oven to occur the polymerization process and form a dried resin. The molar ratio of citric acid to elements (metals + boron) was 3:1. The citric acid/d-sorbitol mass ratio was set to 3:2. The dried resin was calcinated at 400 ^o^C during 24 h, and heat-treated at 700 ^o^C/24 h and at 1100 ^o^C/1 h.

### Nd:YAB crystal optical properties

Nd:YAB crystals forms trigonal structure with space group R32. The lattice parameters are: a = b = 9.293 Å, c = 7.213 Å, Z = 3; the lanthanide ions are substitute for Y^3+^ ions having trigonal prismatic geometry with D_3_ point symmetry and surrounded by six oxygen atoms, the Al^3+^ ions occupy the octahedral sites, and the boron is arranged in sheets of (BO^3^)^3−^ (refs 31 and 32). Nd:YAB exhibit good properties for solid-state lasers: high physical and chemical stability, high thermal conductivity, good mechanical strength^33^. The effective nonlinear optical coefficient, 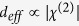, is ~1.40 pm/V which is comparable to that of BBO crystals^34^. They possess thermal conductivity of 0.04 Wcm^−1^K^−1^ and thermal expansion coefficients of the order of 2 × 10^−6^ K^−1^ and 9 × 10^−6^ K^−1^ for *a* and *c* axes, respectively^35^. Moreover, the Nd:YAB crystal is a uniaxial anisotropic medium with the ordinary and extraordinary refractive index of 1.755 and 1.6869, respectively, at 1062 nm, and 1.7808 and 1.7075, respectively, at 531 nm (ref. 36), thus allowing the second-order nonlinear optical processes to take place.

### Optical experiments

All measurements were performed at room temperature. The excitation pulse energy (EPE) was controlled by a pair of polarizers and measured with a calibrated silicon photodiode. The angle between the normal to the sample and the incident beam was 37° and the scattered light was collected from the front surface of the sample by a 5 cm focal length lens. The collected light was focused by a 20 cm lens at the entrance of a spectrometer (nominal resolution: 0.1 nm) equipped with a cooled CCD. The temporal behavior of the emitted light was characterized using a photodetector with a nanosecond response. Optical filters were used to eliminate the excitation laser residue from the collected signal.

## Additional Information

**How to cite this article**: Moura, A. L. *et al.* Multi-wavelength emission through self-induced second-order wave-mixing processes from a Nd^3+^ doped crystalline powder random laser. *Sci. Rep.* **5**, 13816; doi: 10.1038/srep13816 (2015).
